# Design strategy and mechanism of nitrite oxidation suppression of elevated loading rate partial nitritation system

**DOI:** 10.3389/fmicb.2023.1142570

**Published:** 2023-03-29

**Authors:** Juliet Ikem, Huiyu Chen, Robert Delatolla

**Affiliations:** Department of Civil Engineering, Ottawa-Carleton Institute for Environmental Engineering, University of Ottawa, Ottawa, ON, Canada

**Keywords:** municipal wastewater, ammonia removal, anammox, moving bed biofilm reactor, nitrite oxidation suppression, mainstream

## Abstract

There is a current need for a low operational intensity, effective and small footprint system to achieve stable partial nitritation for subsequent anammox treatment at mainstream municipal wastewaters. This research identifies a unique design strategy using an elevated total ammonia nitrogen (TAN) surface area loading rate (SALR) of 5 g TAN/m^2.^d to achieve cost-effective, stable, and elevated rates of partial nitritation in a moving bed biofilm reactor (MBBR) system under mainstream conditions. The elevated loaded partial nitritation MBBR system achieves a TAN surface area removal rate (SARR) of 2.01 ± 0.07 g TAN/m^2.^d and NO_2_^−^-N: NH_4_^+^-N stoichiometric ratio of 1.15:1, which is appropriate for downstream anammox treatment. The elevated TAN SALR design strategy promotes nitrite-oxidizing bacteria (NOB) activity suppression rather than a reduction in NOB population as the reason for the suppression of nitrite oxidation in the mainstream elevated loaded partial nitritation MBBR system. NOB activity is limited at an elevated TAN SALR likely due to thick biofilm embedding the NOB population and competition for dissolved oxygen (DO) with ammonia-oxidizing bacteria for TAN oxidation to nitrite within the biofilm structure, which ultimately limits the uptake of DO by NOB in the system. Therefore, this design strategy offers a cost-effective and efficient alternative for mainstream partial nitritation MBBR systems at water resource recovery facilities.

## 1. Introduction

Partial nitritation and anaerobic ammonia oxidation (anammox), known as the PN/A process, is an autotrophic nitrogen removal process based on two consecutive processes: ammonia-oxidizing bacteria (AOB) oxidizing about 55% of the total ammonia nitrogen (TAN) to nitrite, and subsequently, anammox bacteria (AnAOB) converting the produced nitrite and residual TAN to nitrogen gas with limited nitrate production. Compared to conventional nitrification and denitrification, PN/A is more cost-effective and energy-efficient and could significantly reduce the energy consumption of water resource recovery facilities (WRRFs) by 20–40% (Agrawal et al., 2018; Pedrouso et al., 2019). Over the past decade, the PN/A process has been successfully implemented to treat sludge digester centrate, also termed “sidestream wastewaters,” within municipal WRRFs (Lackner et al., 2014; Lotti et al., 2015b). In recent years, there has been renewed interest in exploring the PN/A process at conditions more typical for mainstream municipal wastewater. However, mainstream municipal wastewaters are characterized by low TAN concentrations (20–40 mg TAN/L), high C/N ratios (7–12 g COD/g-N), variable TAN loading rates, and low temperatures (<10°C), with these characteristics limiting the successful implementation of the PN/A process at WRRFs. These mainstream wastewater characteristics have been demonstrated to limit AOB and AnAOB growth rates and also increase the challenge of achieving effective nitrite oxidation suppression; which is necessary to maintain ideal effluent quality and long-term process stability in mainstream PN/A systems (Gilbert et al., 2015; Li et al., 2018; Hoekstra et al., 2019).

Long-term nitrite oxidation suppression in mainstream PN/A systems has been investigated using a one-stage system, where partial nitritation and anammox occur in the same reactor, or a two-stage system with partial nitritation and anammox occurring in separate reactors (Laureni et al., 2016; Chen et al., 2018; Gu et al., 2018; Gustavsson et al., 2020; Schraa et al., 2020; Trojanowicz et al., 2021; Choi and Jung, 2022). While the one-stage configured systems are mostly studied due to the lower cost of infrastructure than two-stage configured systems, the latter offers the opportunity to optimize the stable partial nitritation and anammox process independently and maintain a balanced ratio between functional bacteria groups (AOB and AnAOB) to achieve higher volumetric nitrogen removal rates.

In a two-stage configured system, optimization of the partial nitritation process requires effective operational control strategies to ensure that the nitrite-oxidizing bacteria (NOB) population or activity is continuously suppressed within the system (Piculell et al., 2016a; Gu et al., 2018; Choi and Jung, 2022). In this regard, several operational control strategies have been employed in a two-stage configured system involving either suspended growth or attached growth systems to achieve stable partial nitritation. Recently, research has focused on hybrid system where attached growth biofilms and flocs coexist, also known as integrated fixed-film activated sludge (IFAS) systems, and biofilm technologies such as the moving bed biofilm reactor (MBBR) system (Laureni et al., 2016, 2019; Gu et al., 2018; Kowalski et al., 2019a; Gustavsson et al., 2020; Trojanowicz et al., 2021). Recent interest in biofilm technologies is due to their ability to retain highly specialized microbial populations and also because biofilm structure results in substrate gradients that can promote suppression of the growth of the NOB population or suppression of the NOB activity (Brockmann and Morgenroth, 2008; Gilbert et al., 2014; Pérez et al., 2014; Lotti et al., 2015a; Piculell et al., 2016a).

Several operational control strategies have been applied using the MBBR technology to selectively inhibit NOB population or activity (Lotti et al., 2014; Pérez et al., 2014; Gilbert et al., 2015; Laureni et al., 2016). Some of the operational control strategies that have been reported in mainstream partial nitritation two-stage MBBR systems include: alternating feed from sidestream to mainstream; bioaugmentating with AOB biomass; controlling biofilm thickness; exposure of biomass to toxic sidestream effluent or conditions favorable for AOB growth; employing a combination of dissolved oxygen (DO)/TAN ratio control and free ammonia (FA) inhibition; and alternating anoxic and aerobic condition through intermittent aeration (Piculell et al., 2016a,b; Gu et al., 2018; Kowalski et al., 2019a,b). Notwithstanding the potential of NOB suppression using these operational control strategies, these strategies are all operationally intensive and have demonstrated difficulty in achieving long-term suppression of NOB population or activity, overall process stability, and stable effluent quality.

In recent studies, an elevated TAN loading rate, as a means of a passive and low operational design strategy, has been shown to achieve stable partial nitritation rates within a two-stage configured MBBR system (Schopf et al., 2019, 2021; Ikem et al., 2023). Schopf et al. (2019, 2021) achieved stable and robust partial nitritation at an elevated TAN loading rate of 6.5 g TAN/m^2.^d in an MBBR system fed with TAN concentration of 125mg TAN/L, simulating TAN concentrations observed in industrial wastewaters. In this system, stable partial nitritation was attributed to the morphological impacts resulting from operating at an elevated TAN loading rate that could allow for NOB activity to be effectively suppressed (Schopf et al., 2021). Consequently, the feasibility of using this design strategy to achieve stable partial nitritation has been evaluated at conditions and TAN concentrations between 25 to 44 mg TAN/L typical for mainstream municipal wastewater (Ikem et al., 2023). Ikem et al. identified critical design parameters, a TAN surface area loading rate (SALR), a hydraulic retention time (HRT), and an airflow rate optimized for stable partial nitritation of a mainstream elevated loaded MBBR system. However, this system did not achieve the ideal effluent NO_2_^−^-N:NH_4_^+^-N stoichiometric ratio of 1.31:1 for subsequent anammox treatment. Specifically, the average NO_2_^−^-N:NH_4_^+^-N stoichiometric ratio reported in this study was at 0.70:1, which is not comparable to the optimized ratio proposed by Strous et al. (1998) for anammox operation. The operation of anammox system with inappropriate effluent stoichiometric ratio from the partial nitritation system has been demonstrated to impact the attachment and enrichment of anammox cells in a full-scale MBBR, leading to process instability (Stefansdottir et al., 2015). Hence, further optimization of the elevated loaded partial nitritation MBBR system is required to improve effluent quality for downstream anammox operation. Moreover, further investigation of biofilm characteristics, embedded cells, and microbiome response is needed to understand nitrite oxidation suppression mechanism responsible for stable partial nitritation of mainstream elevated loaded MBBR systems. Although previous work has investigated the mechanism and method of nitrite oxidation suppression caused by employing elevated TAN loading rate for partial nitritation control, these studies were performed at higher influent TAN concentrations not typically observed in mainstream municipal wastewater (Schopf et al., 2019, 2021). Thus, there is a significant knowledge gap and a need to investigate further the mechanism of nitrite oxidation suppression in the elevated loaded partial nitritation system that would ensure high performance and long-term operational stability under mainstream conditions.

Therefore, this study aims to identify the mechanism of nitrite oxidation suppression of mainstream elevated loaded partial nitritation MBBR systems. The specific objectives were to (i) characterize the performance of a two-reactor in series designed mainstream elevated loaded partial nitritation MBBR system; (ii) determine the effects of elevated TAN loading rate on biofilm thickness, biofilm mass, biofilm density, and embedded cells, and how these characteristics influence the performance of the mainstream partial nitritation MBBR system; and (iii) quantitate the AOB and NOB population counts within the attached growth, biofilm community and identify whether NOB population suppression or NOB activity suppression is responsible for partial nitritation.

## 2. Materials and methods

### 2.1. Reactor configuration and operation

**Figure 1:**
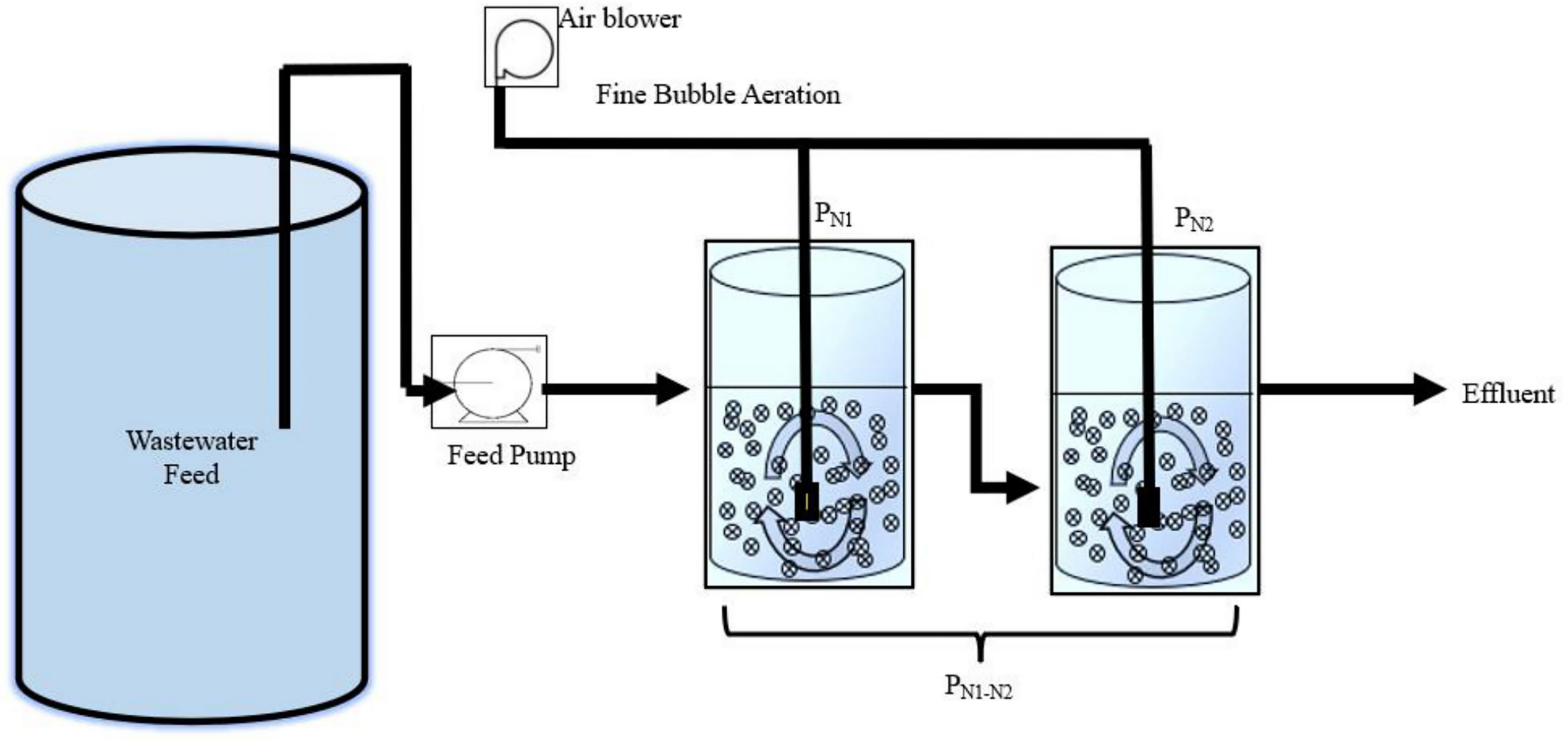
Experimental set-up.

Two-reactors in series partial nitritation MBBR system configuration were identified in the preliminary work of this study based on findings from Ikem et al. (2023) to optimize the effluent concentration from the partial nitritation MBBR system for subsequent downstream anammox treatment. The experimental set-up consisted of two identical lab-scale MBBR reactors (2L each) operated in series (Figure 1). The reactors were filled with high-density polyethylene AnoxK™5 carriers (AnoxKaldnes, Lund, Sweden) with a diameter of 25 mm, height of 4 mm, and bulk surface area of 800 m^2^/m^3^. The AnoxK™5 were seeded carriers harvested from a biological oxygen demand (BOD) removal municipal IFAS wastewater treatment system located in Hawkesbury, Ontario, Canada, and were operated in a single bench lab partial nitritation MBBR system operated at elevated TAN concentrations prior to this study. The reactors were designed at a target TAN SALR of 5 g TAN/m^2.^d, recommended as the optimum for the partial nitritation MBBR system based on the previous findings, as it demonstrated stable and steady performance as well as high partial nitritation rate (Schopf et al., 2019; Ikem et al., 2023). The TAN SALR in the nitrifying MBBR systems at ambient temperature are conventionally designed typically between 0.45 to 1.0 g TAN/m^2.^d with respect to target effluent TAN concentrations (Hem et al., 1994; Odegaard, 1999; Young et al., 2017b); thus, the TAN SALR present in this study was significantly elevated (referred to as elevated TAN SALR) than conventional TAN SALR which would potentially allow operating high-rate partial nitritation and small land footprint system. The elevated TAN SALR of 5 g TAN/m^2.^d in the partial nitritation reactors was achieved at mainstream concentrations through operation at relatively short target HRT of 2 h and low carrier fill fraction, at 6 and 9.5%. The first reactor, denoted P_N1,_ contained 39 carriers, and the second reactor P_N2_, contained 28 carriers. The carriers accounted for 9.5 and 6% of the total reactor volume, respectively. These different fractions were necessary to operate both reactors at similar elevated TAN SALR values of 5 g TAN/m^2.^d, as P_N2_ was fed with the effluent of P_N1_ and hence required fewer carriers to achieve the same SALR as P_N1_. For clarity and easy understanding of this manuscript, the reactors in a series of P_N1_ and P_N2_ will be referred to as P_N1-N2_.

P_N1_ and P_N2_ were both equipped with a coarse bubble aeration system, with diffusers positioned at the base of the reactors to provide adequate DO in the bulk solution and allow sufficient mixing of the carriers. The airflow rates were maintained at 1.5 and 1L/min for P_N1_ and P_N2_, respectively, as previous findings demonstrated that adjusting airflow above 1.5 L/min resulted in increased nitrite oxidation to nitrate (Ikem et al., 2023). Moreover, P_N2_ had lower influent TAN concentration and fewer carriers than P_N1_; as such would necessitate a reduced airflow rate to limit nitrite oxidation. P_N1_ was fed with a peristaltic pump with influent from a wastewater feed tank, and P_N2_ was fed *via* gravitational flow with the effluent from P_N1_. The target (measured) influent TAN concentrations were 30 (32.16 ± 0.46) mg TAN/L for P_N1_ and 20 (20.4 ± 1.54) mg TAN/L for P_N2_, all within the conventional limits of mainstream municipal TAN concentrations. The operational conditions for both reactors were the same with the following target (measured) values: SALR of 5 (5.07 ± 0.22) g TAN/m^2.^d, HRT of 2 (2.02 ± 0.02) h, DO of 6.5 (6.84 ± 0.05) mg O_2_ /L, pH of 7.5 (7.76 ± 0.05), and temperature 20 (19.9 ± 0.10)^o^C.

### 2.2. Wastewater feed

Synthetic wastewater (SWW) was prepared based on a recipe from previous studies (Delatolla et al., 2009; Schopf et al., 2019; Tian et al., 2019). The SWW simulated wastewater effluent from a carbon removal process without TAN removal and was composed as follows (per L of SWW): 0.14 g (NH_4_^+^)_2_SO_4_ (corresponding to a concentration of approximately 30 mg NH_4_^+^-N/L), 0.39 g NaHCO_3_, 0.06 g MgSO_4_· 7H_2_O, 0.02 g CaCl_2_·2H_2_O, 0.06 g KH_2_PO_4_, and 0.004 g FeSO_4_·7H_2_O. Trace nutrients (per L of SWW) included: MnCl_2_·4H_2_O: 0.10 mg, Na_2_MoO_4_·2H_2_O: 0.03 mg, CuSO_4_·5H_2_O: 0.10 mg, CoCl_2_·6H_2_O: 0.001 mg, and ZnSO_4_·7H_2_O: 0.03 mg. The carbon source composition (per L of synthetic wastewater): glucose 4.86 mg, sodium acetate 2.59 mg, and peptone 4.86 mg resulted in a sCOD concentration of 25 mg sCOD/L, thereby mimicking post-carbon effluent (Schopf et al., 2019).

### 2.3. Constituent analyses

Wastewater constituent analyses were performed on each of the two MBBR reactors in series, P_N1_ and P_N2_. Wastewater samples were collected three times a week from both reactors for analyses, and the samples were tested in triplicate for the following parameters: TAN, nitrite, nitrate, DO, sCOD, pH, temperature, alkalinity, total suspended solids (TSS), and volatile suspended solids (VSS). To quantify the kinetics of the reactors, the following standard methods were used: Nessler-4500C-NH_3_, 4500B-NO_2_^−^ and nitrate 4500A-NO_3_^−^ for TAN, nitrite, and nitrate, respectively. DO and temperature measurements were acquired using a HACH Flexi HQ30d DO/temperature probe (HACH, Loveland, CO, United States) and pH was measured using a SympHony VWR pH probe (VWR, Ontario, Canada). sCOD was quantified using a HACH 8000 (HACH, Loveland, CO, United States), and TSS and VSS were measured using standard methods 2540D (Clesceri et al., 1999). In addition, the aeration rates were monitored and measured using a Dwyer VFA-24 Visi-Float® acrylic airflow meter (DWYER, Michigan City, IN, USA).

### 2.4. Biofilm morphology, thickness, and mass

Stereomicroscopy was used to acquire images of *in-situ* biofilm thickness and morphology. Stereomicroscopy was used because it does not require any sample preparation as such maximizes the integrity of the sample and artifact creation. Four carriers per reactor were extracted and analyzed within 4 h of being harvested. Five images per carrier were acquired at ×2 magnification and were used to determine biofilm thickness. Also, one image per carrier was acquired and analyzed at ×4 magnification to assess the biofilm surface morphology. All of these images were taken at random locations across the carrier surface to avoid bias. Finally, two images per carrier were obtained at ×0.8 magnification to verify biofilm attachment across the carriers. The biofilm thicknesses were quantified using ImageJ 1.52a image processing software (Wayne Rasband, USA).

Biofilm mass was measured using a protocol described and modified by Delatolla et al. (2008), Ren et al. (2016), Schopf et al. (2018), and Arabgol et al. (2020). Biofilm carriers were harvested from MBBR reactors P_N1_ and P_N2_ and dried at 105°C overnight. Dried carriers were cooled in a desiccator for a minimum of 30 min, after which their weights were recorded as W_1_. The dried carriers were then thoroughly cleaned with a stiff-bristled brush and warm water and were dried again at 105°C overnight. The dried carriers were cooled for another 30 min in a desiccator, and their weights were recorded as W_2_. The biofilm mass was calculated as the difference between W_1_ and W_2_. Prior testing was done to ensure that biofilm removal and heating did not cause any significant change in the mass of the biofilm carrier.

### 2.5. Cell viability

Biofilm carriers were harvested from each reactor P_N1_ and P_N2_ and cut into sections to expose the inner biofilm surfaces. The sections were stained using a Film Tracer LIVE/DEAD biofilm viability kit (Life Technologies Ontario, Canada), which comprises SYTO 9 stain (a green nucleic acid stain that illuminates the intact cell membrane) and propidium iodide, which only stains cells with damaged cell membranes (i.e., non-viable cells). Calcofluor white stain (Sigma-Aldrich, MO, United States) was used to fluoresce biofilm extracellular polymeric substances (EPS) (Chen et al., 2007). Viable and non-viable embedded cells in the biofilm were observed using a 510/AxioImager confocal laser scanning microscope (CLSM) (Zeiss, VA, United States) equipped with argon and helium-neon lasers with a variety of wavelengths. A minimum of 20 images were acquired at over a depth of 50 μm to determine the number of viable and nonviable cells across the entire biofilm depth. Cell viability based on the CLSM images was determined using NI Vision Assistant 8.0 software (National Instruments, LabView, 2018). The biofilm area was determined and outlined by tracing the calcofluor white stain. The image color threshold on the CLSM images was used to calibrate the area of an identifiable single cell. The standardized images were then used to quantify the biofilm area and the area of viable and non-viable cells (Delatolla et al., 2009; Ren et al., 2016; Ahmed and Delatolla, 2021).

### 2.6. Quantification of AOB and NOB

#### 2.6.1. DNA extraction

Ammonia-oxidizing bacteria and NOB cell counts were determined using droplet digital PCR (ddPCR). Two carriers each, from reactors P_N1_ and P_N2,_ were collected, and genomic DNA from their biofilms was extracted using a FastDNA*Spin Kit for Soil (MP Biomedicals, CA, United States). The DNA concentrations were analyzed using an Invitrogen Qubit™ 3.0 Fluorometer. Extracted DNA was stored at –80°C until ddPCR analysis.

#### 2.6.2. ddPCR analyses

AOB cell counts were quantified by targeting ammonia monooxygenase subunit A (amoA). The set of amoA primers (*amoA*-1f/*amoA*-2r) targeted a stretch of conserved regions of the known amoA gene sequence of *Nitrosomonas europaea*. Also, two coupled primers, FGPS872f/FGPS1269r and NSR1113f/NSR1264r, were used to amplify the NOB, *Nitrobacter,* and *Nitrospria,* respectively (Table 1; Degrange and Bardin, 1995; Rotthauwe et al., 1997; Geets et al., 2007). The ddPCR mixture was constituted using 5L of sample and 11L of QX200™ ddPCR™ EvaGreen*Supermix (Bio-Ras, Hercules, CA, United States), including 0.23 L of each forward and reverse primer (10 mol/L) and 6.04 L of nuclease-free water. Each ddPCR analysis was performed using a 20 L reaction mixture combined with 65 L of droplet generation oil for EvaGreen to form droplets using a droplet generator. Using a multi-channeled pipette, 40 L of generated droplets were carefully transferred into a 96 well ddPCR plate (Eppendorf, Hamburg, Germany). The plate was sealed using a plate sealer machine and transferred into a T100™ thermal cycler (Bio-Rad, Hercules, CA, United States) for DNA amplification. The amplification program consisted of the following steps: denaturation at 95°C for 5 min, followed by 50 cycles of 30 s at 95°C, 60 s at the corresponding primer annealing temperature (Table 1), and 30 s at 72°C, followed by a 5 min cooldown at 4°C, 5 min at 90°C for droplet stabilization and a hold at 12°C. The QX200 droplet reader (Bio-Rad, Hercules, CA, United States) was applied, and the results were analyzed using QuantaSoft Software (Bio-Rad, version 1.7.4, Hercules. CA, United States). The software measures the number of positive and negative droplets per fluorophore per sample, with each positive counted as a 1 and each negative counted as a 0. The quality control measure ensured that the total droplets quantified were >10,000, and <5 positive droplets were identified as negative controls (Tian et al., 2020).

**Table 1:** Primer details and annealing temperatures.

Target	Primer	Sequence (5′-3′)	Annealing temperature (°C)	Reference
AOB	amoA-1f	GGGGTTTCTACTGGTGGT	53	Rotthauwe et al. (1997)
	amoA-2r	CCCCTCKGSAAAGCCTTCTTC		
NOB – *Nitrobacter*	FGPS872f	CTAAAACTCAAAGGAATTGA	56	Degrange and Bardin (1995)
	FGPS1269r	TTTTTTGAGATTTGCTAG		
NOB – *Nitrospira*	NSR1113f	CCTGCTTTCAGTTGCTACCG	53	Bao et al. (2017)
	NSR1264r	GTTTGCAGCGCTTTGTACCG		

### 2.7. Statistical analyses

Statistical analyses of wastewater constituents, biofilm thickness, mass and viable/non-viable cell percentages were determined using the student’s *t*-test, with a *value of p* less than 0.05 indicating a significant difference. In addition, the student’s *t*-test was used to ascertain statistical significance for the bacterial gene copies with a *value of p* of less than 0.10 considered to indicate a statistically significant difference. Graphical charts and bars were prepared using GraphPad Prism 8.4.1, with error bars in figures being plotted at the 95% confidence intervals (CI).

## 3. Results and discussion

### 3.1. Performance of a two-reactor in series partial nitritation MBBR system

**Figure 2:**
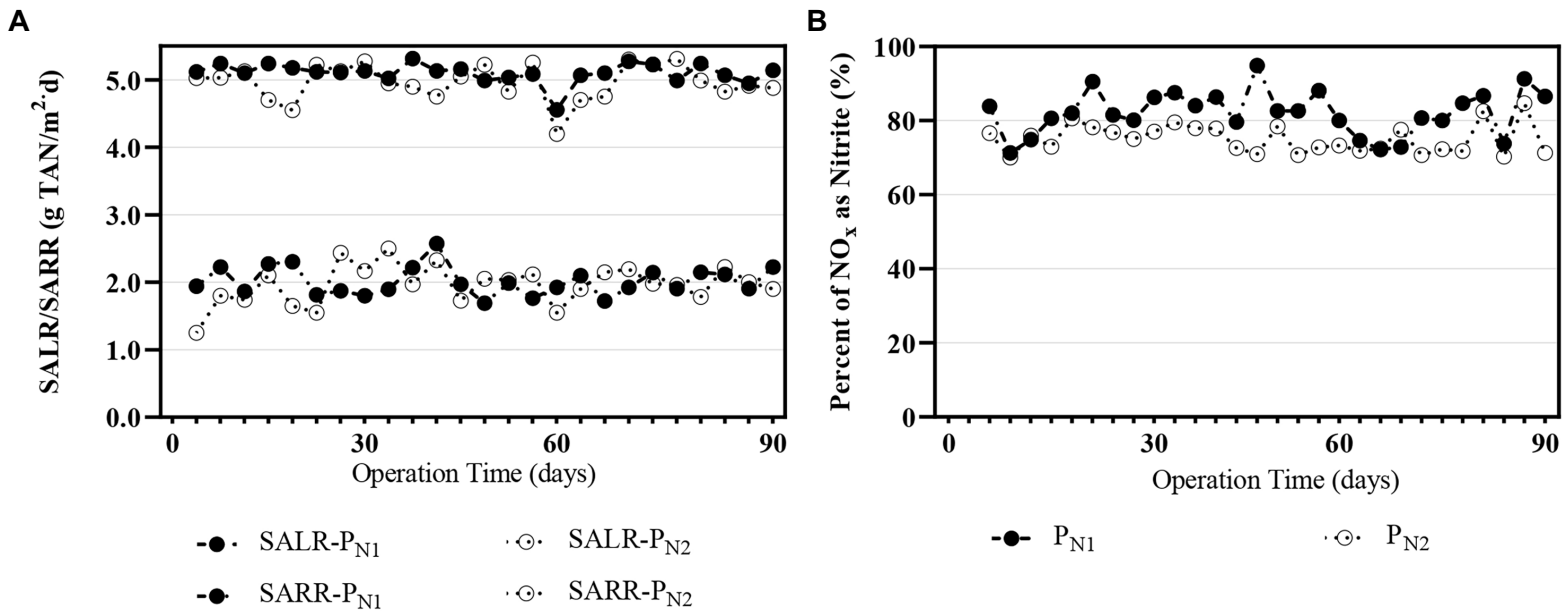
Performance of partial nitritation MBBR system **(A)** SALR/SARR over time P_N1_ and P_N2_ **(B)** percent NO_x_ as nitrite over time for P_N1_ and P_N2._

The partial nitritation MBBR system, both P_N1_ and P_N2_, was operated at a target elevated TAN SALR of 5 g TAN/m^2.^d and showed steady and stable performance over a 90-day period of operation (Figure 2), with measured SALR values for P_N1_ and P_N2_ 5.07 ± 0.22 and 5.05 ± 0.12 g TAN/m^2.^d, respectively. The average TAN surface area removal rate (SARR) of the first reactor, P_N1,_ is 2.25 ± 0.08 g TAN/m^2.^d, corresponding to a TAN oxidation efficiency of 45.3 ± 1.1%. The resultant TAN SARR of 2.01 ± 0.07 g TAN/m^2.^d from the complete partial nitritation MBBR in series system, P_N1-N2_, (Figure 2A), corresponds to a TAN oxidation efficiency of 59.7 ± 1.3%. These values are comparable to the ideal TAN oxidation efficiency of 57%, based on the stoichiometric NO_2_^−^-N: NH_4_^+^-N molar ratio of 1.32:1 for subsequent anammox treatment (Strous et al., 1997; Van Dongen et al., 2001). The observed TAN oxidation efficiency from the partial nitritation MBBR system, P_N1-N2,_ is also similar to the TAN oxidation efficiency of 60% previously reported in the mainstream partial nitritation MBBR system while utilizing a combination of DO/TAN ratio control and FA inhibition as control strategies (Kowalski et al., 2019b). However, the current elevated loading, two-reactor in series design strategy herein studied does not require operational control while demonstrating robust and steady performance.

The percent NO_x_ as nitrite was monitored in the partial nitritation MBBR system over a period of 90 days and demonstrates stable and steady performance (Figure 2B). The P_N1_ and P_N2_ reactors show an average percent NO_x_ as nitrite of 81.2 ± 2.1 and 74.7 ± 0.9%, respectively. The average percent NO_x_ as nitrite of the total oxidized TAN from the partial nitritation MBBR system, P_N1-N2_, indicates that partial nitritation was achieved, i.e., TAN was oxidized to nitrite and oxidation of nitrite to nitrate was largely mitigated. The relative nitrate production varied between 13 and 16% in the reactors P_N1_ and P_N2_. Similar levels of relative nitrate production have also been reported in mainstream partial nitritation and single-stage PN/A MBBR systems (Gilbert et al., 2014; Persson et al., 2017; Gu et al., 2018; Gustavsson et al., 2020). Therefore, the minimal levels of nitrate build-up in the elevated loaded partial nitritation MBBR system suggest that the NOB population in the biofilm or the NOB activity of the embedded population are possibly suppressed.

**Figure 3:**
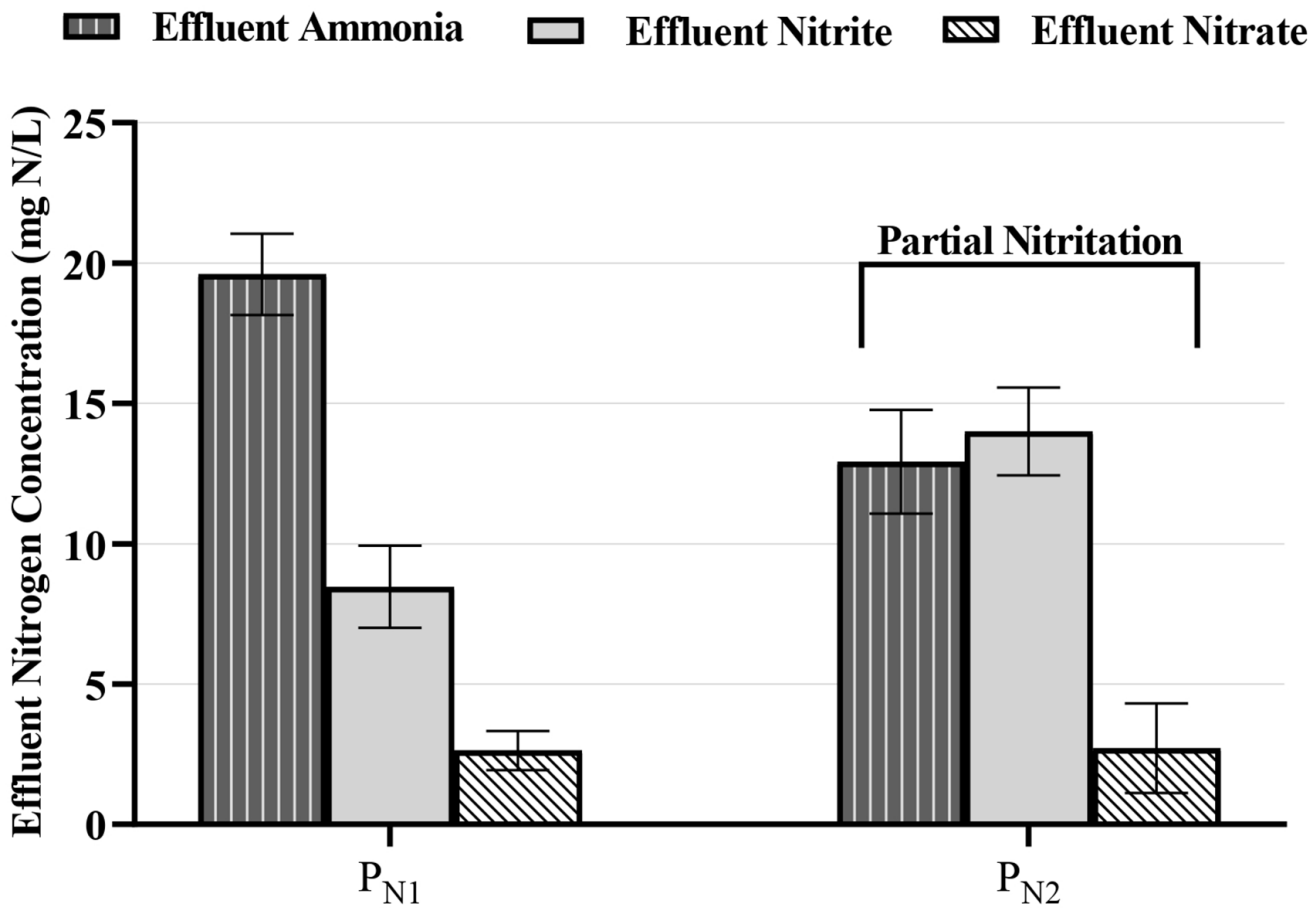
Effluent TAN (vertical shading), nitrite (solid light gray fill), and nitrate (diagonal shading) concentrations in the partial nitritation MBBR system, P_N1_ and P_N2_ (average ± 95%CI).

Finally, the average effluent TAN, nitrite, and nitrate concentrations from reactor P_N1_ of 20.1 ± 1.30 mg TAN/L, 7.14 ± 0.81 mg NO_2_^−^-N/L, and 4.02 ± 0.21mg NO_3_^−^-N/L demonstrate a NO_2_^−^-N:NH_4_^+^-N stoichiometric ratio of 0.36:1 (Figure 3). This stoichiometric ratio from reactor P_N1_ is below the ideal metabolic ratio of 1.32:1 for partial nitritation systems upstream of anammox systems. From a practical standpoint, a stoichiometric ratio near 1:1 has also been reported sufficient for mainstream anammox operation in an MBBR system (Gu et al., 2018). Therefore, with the introduction of the second reactor (P_N2_) in series to the first reactor (P_N1_) in this study, the average effluent TAN, nitrite, and nitrate concentrations of 12.9 ± 1.84 mg TAN/L, 13.9 ± 1.56 mg NO_2_^−^-N/L and 3.11 ± 0.29 mg NO_3_^−^-N/L, were measured respectively. These P_N2_ effluent concentrations correspond to a metabolic ratio of 1.15:1. As such, this ratio provides suitable nitrogen speciation for the subsequent downstream anammox treatment, and the two-reactors in series partial nitritation MBBR system configuration in this study herein provides a stable effluent quality for subsequent downstream anammox operation.

### 3.2. Biofilm characteristics and morphology

**Figure 4:**
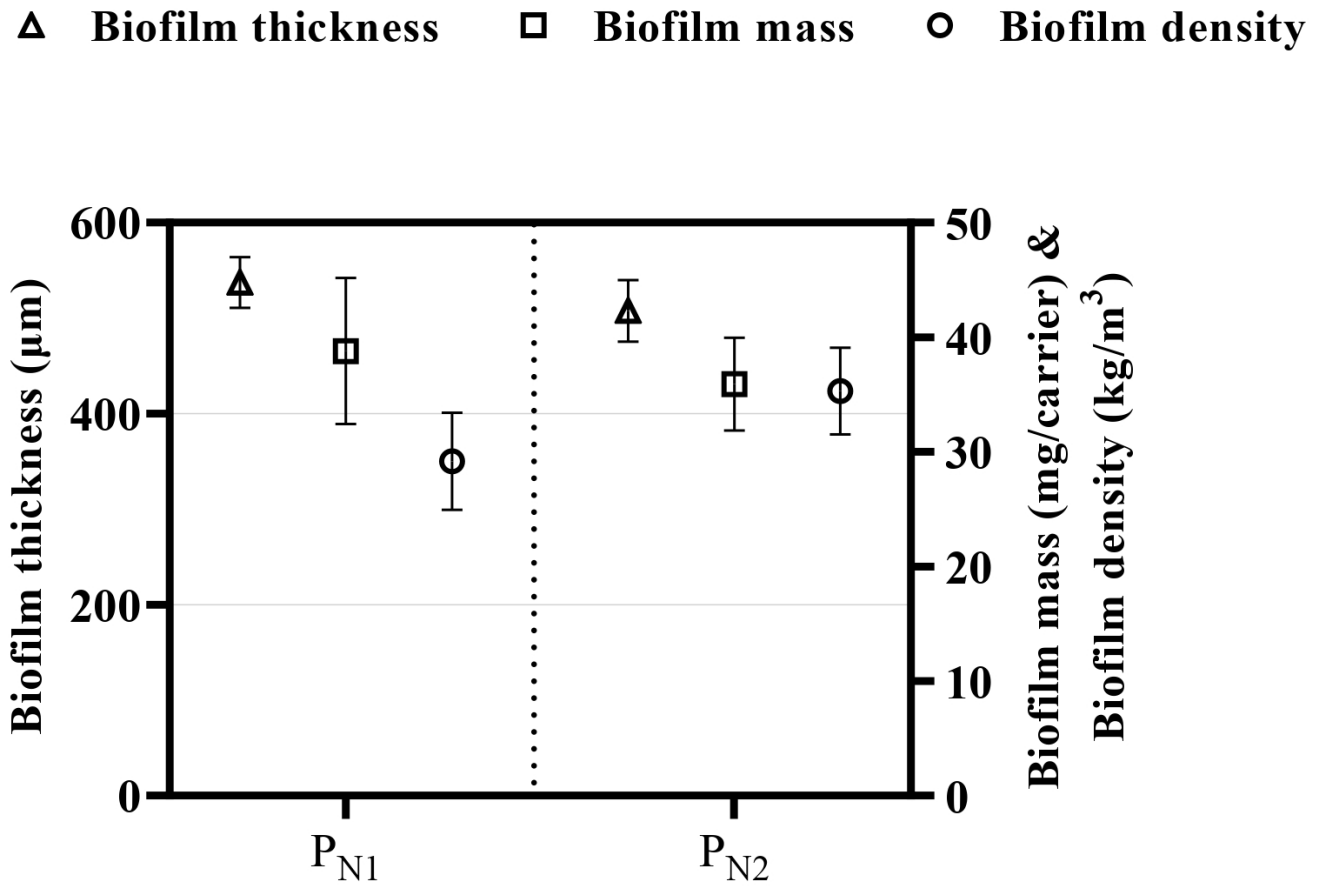
Biofilm thickness (triangle), biofilm mass (square), and biofilm density (circle), for partial nitritation MBBR system, P_N1_, and P_N2_ (average ± 95% CI).

The meso-scale effects of the elevated TAN SALR on the biofilm were quantified through the analysis of biofilm characteristics described as biofilm thickness, biofilm mass, biofilm density, and morphology (Figure 4). The biofilm thickness is 577 ± 21 and 517 ± 43 μm; the biofilm mass is 38.8 ± 1.2 and 35.5 ± 3.1 mg/carrier corresponding to biofilm density (calculated based on biofilm thickness and biofilm mass) of 29.9 ± 9.2 and 33.7 ± 8.7 kg/m^3^ in the P_N1_ and P_N2_ reactors, respectively. The average biofilm thickness of 547 ± 65 μm in the partial nitritation MBBR system (P_N1_ and P_N2_) is higher than the 300 μm reported in the mainstream partial nitritation MBBR system (Piculell et al., 2016b,c). The difference in biofilm thickness could be expected as the carrier type and control strategy utilized in both studies are different and would result in varying biofilm composition or characteristics. However, the biofilm thickness, mass, and density are within the range reported in a previous study on elevated loaded partial nitritation MBBR systems with comparable operational conditions (Schopf et al., 2021).

On the other hand, the qualitative assessment of the biofilm shape and morphology shows a rough, irregular, and thick biofilm. This finding is consistent with previous studies showing thick biofilms at similar elevated TAN SALR of 5 g TAN/m^2.^d in a partial nitritation MBBR system (Schopf et al., 2019). The thick biofilm is possible as elevated loading conditions and high substrate load allow for a deeper substrate penetration into the biofilm, supporting high cell growth. Furthermore, previous studies have demonstrated that increased biofilm thickness leads to steep substrate gradients and stratification of the metabolic process throughout the biofilm, likely resulting in a more biodiverse and heterogeneous biofilm, thus potentially influencing the overall performance of the system (Torresi et al., 2016; Suarez et al., 2019). Therefore, the stable and steady partial nitritation performance observed in this study at elevated TAN SALR could be explained by stratification that occurs in thick biofilms with AOB population dominating the upper layers of the biofilm and NOB population at deeper layers as the system operates under DO mass transfer rate limited conditions (Pérez et al., 2014; Malovanyy et al., 2015). As such, NOB population or activity is likely suppressed due to the limited diffusion of DO in the biofilm of elevated loading partial nitritation MBBR system of the herein study.

### 3.3. Cell viability

**Figure 5:**
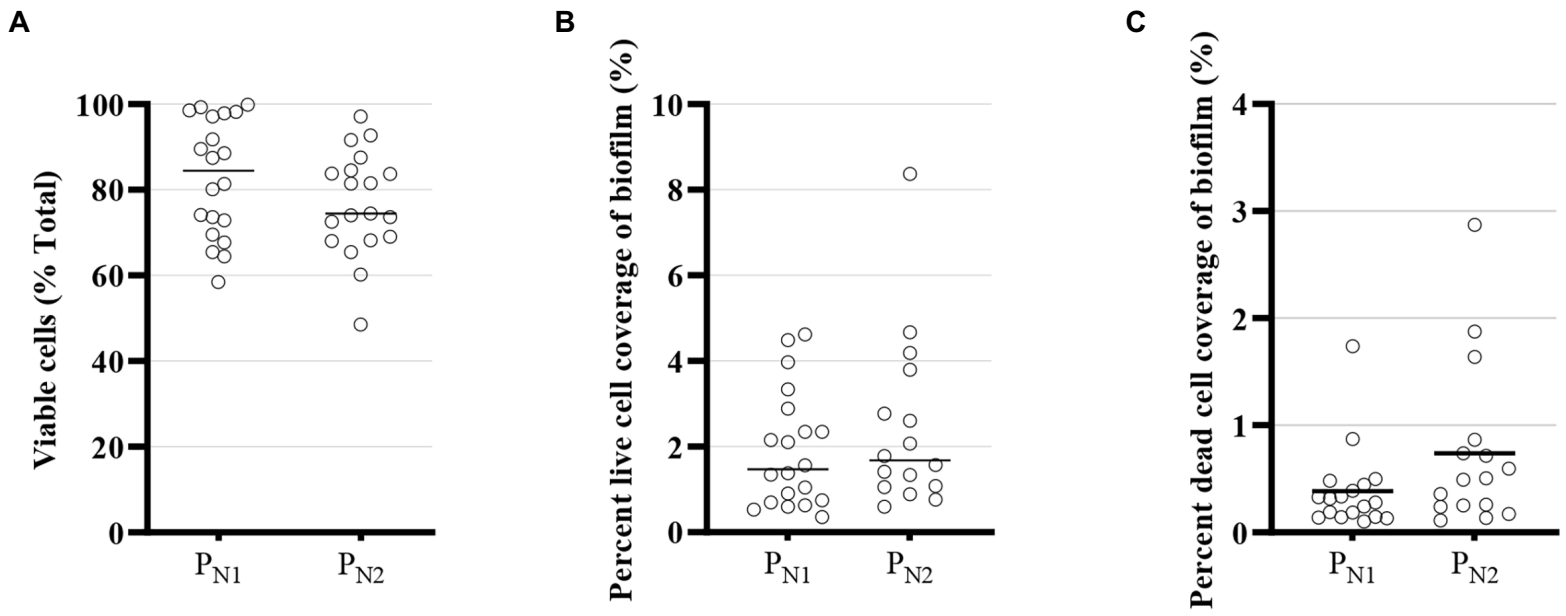
Confocal laser scanning microscope (CLSM) viability of embedded cells for partial nitritation MBBR system, P_N1_ and P_N2_: **(A)** viability as a fraction of total cells (%) **(B)** percent live-cell coverage of biofilm, and **(C)** percent dead cell coverage (average ± 95% CI).

In an MBBR system, the higher substrate removal rate can be related to live cells or biomass activities on the carrier (Ødegaard, 2006), and as such, the biomass embedded in the biofilm controls the reactor performance. Biofilm structure, in general, and within the MBBR system, typically consists of viable cells and non-viable bacterial cells (Figure 5) along with numerous other substances secreted by the cells and attached substances to the extracellular matrix of the biofilm. The total cell viability (%) ranges from 58.5 to 99.9% in P_N1_ and from 45.8 to 97.1% in the P_N2_ reactor (Figure 5A). Although total cell viability is likely affected by residual TAN concentration and specific embedded microbial communities, the live fraction of total cells, which is the ratio of live cells and the total number of cells (viable + non-viable cells), is stable (Figures 5B,C) and within conventional range in a nitrifying MBBR system (Young et al., 2017a). Hence, the viability of the cells does not appear to constrain the performance of the elevated loaded partial nitritation MBBR reactors, and the mainstream elevated loaded partial nitritation MBBR system retained carriers with viable embedded biomass to maintain sufficient microbial activities for stable and steady partial nitritation performance.

### 3.4. Quantification of AOB and NOB

**Figure 6:**
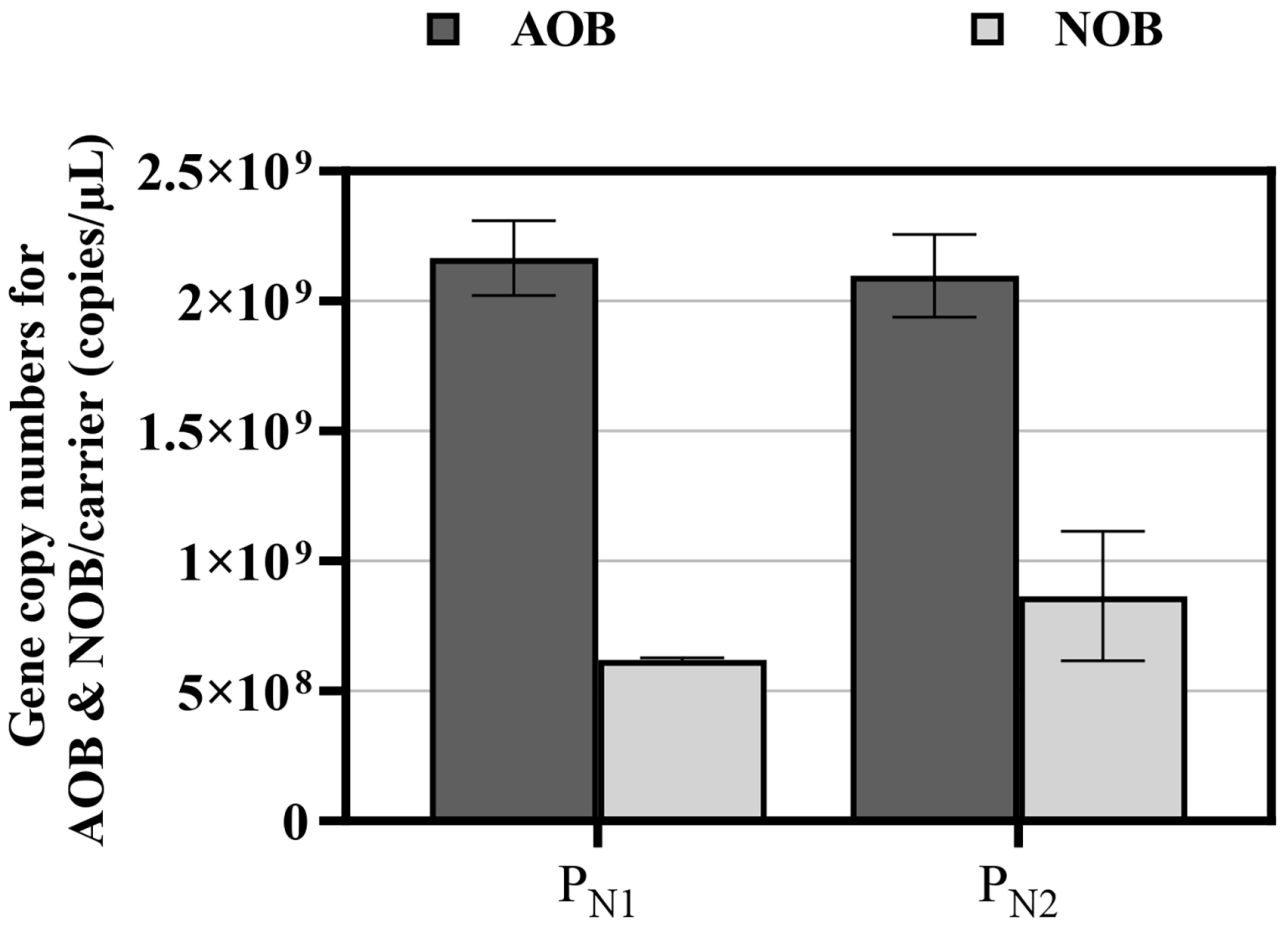
ddPCR data for partial nitritation MBBR system P_N1_ and P_N2_, showing AOB (dark gray fill) and NOB (light gray fill) gene copies (average ± 95% CI).

The copies of AOB and NOB were quantified in the partial nitritation MBBR reactors P_N1_ and P_N2_ using ddPCR (Figure 6). In mainstream partial nitritation reactors, the low bulk nitrite concentration would likely select for *Nitrospira* rather than *Nitrobacter* due to its high substrate affinity (Wang et al., 2019). As such, *Nitrospira* was the only NOB detected in the reactors P_N1_ and P_N2_. During the stable and steady partial nitritation, the average gene copy numbers of AOB are 1.92 ± 3.40 × 10^9^ and 2.02 ± 2.51 × 10^9^ copies/carrier, and the average gene copy numbers of NOB are 6.20 ± 1.55 × 10^8^ and 1.15 ± 1.20 × 10^9^ copies/carrier, in reactor P_N1_ and P_N2_, respectively. These average AOB and NOB, gene copy numbers are statistically similar and correspond to AOB to NOB ratios of 3.21:1 and 2.12:1 in reactors P_N1_ and P_N2_, respectively. These low ratios are comparable to a single mainstream PN/A MBBR system (Gilbert et al., 2015). Also, these values are higher than those published from full nitrification conventional suspended growth systems by Limpiyakorn et al. (2005) and Yao and Peng (2017), and full nitrification MBBR system by Zhang et al. (2019). However, these AOB to NOB ratios are lower than those reported in partial nitritation, conventional suspended growth, and MBBR systems operated under elevated TAN concentrations (Abzazou et al., 2016; Zhang et al., 2020). This distinction in AOB to NOB ratios in full nitrification and partial nitritation systems is possible due to varying reactor types and experimental and operational conditions. Therefore, in the study, the elevated loaded partial nitritation MBBR system demonstrates that NOB activity suppression is the likely dominant mechanism responsible for stable and steady partial nitritation performance under mainstream conditions.

### 3.5. The mechanism of nitrite oxidation suppression in the mainstream partial nitritation MBBR system

To determine the mechanism of nitrite oxidation suppression caused by the low operational intensity partial nitritation design strategy, the driving hypothesis is that elevated TAN SALR and the resulting impact on the biofilm morphology will play a key role in suppressing NOB population or activity in the partial nitritation MBBR system. Previous studies have suggested that the steep substrate gradient present in the biofilm and competition for DO between AOB and NOB, acts as the main mechanism for the control of NOB population or activity in a biofilm-based system (Brockmann and Morgenroth, 2008; Pérez et al., 2014; Isanta et al., 2015; Laureni et al., 2016). This is possible as external mass transfer boundary layer controls the movement of the substrate (TAN) or the electron acceptor (DO) and determines the actual TAN or DO concentration at the biofilm surface (Brockmann and Morgenroth, 2008; Pérez et al., 2014; Isanta et al., 2015; Laureni et al., 2016). The resultant mass transfer limitations (DO mass transfer limited condition) have been numerically demonstrated to promote the suppression of NOB in a partial nitritation MBBR system (Pérez et al., 2020).

Therefore, in this study, elevated TAN SALR and resulting morphological impact on biofilm is the main factor that could be possibly controlling NOB activity suppression rather than NOB population suppression. At elevated TAN SALR, AOB possibly has a higher competitive advantage over NOB due to the high TAN concentration in the bulk solution resulting from elevated TAN SALR, which results in DO being preferentially consumed by AOB compared to NOB. Moreover, elevated TAN SALR, in addition to the resultant biofilm thickness and morphology (roughness), builds an increased mass transfer resistance that possibly limits the mass transfer of oxygen from the bulk solution into the biofilm, resulting in limited DO uptake by NOB and subsequently suppressing their activity. Hence, by maintaining significant AOB activity and suppressing NOB activity, the MBBR system achieves 59.7% TAN oxidation efficiency and, thus, a NO_2_^−^-N:NH_4_^+^-N stoichiometric ratio of 1.15:1 with 83.2 ± 1.2% percent of NO_x_ as nitrite.

### 3.6. The implications of the study

The study shows that an elevated loading design strategy, as opposed to operational control strategies, is able to achieve stable, steady, and robust partial nitritation in an MBBR system under mainstream municipal conditions. The finding from this study provides a feasible design strategy that does not require multiple and complex operational control measures or monitoring, to achieve stable and steady partial nitritation. The operation at elevated TAN SALR and the resulting impact on biofilm morphology essentially restricts DO uptake by NOB, which is beneficial to the long-term suppression of their activity. Therefore, elevated TAN SALR allows for effective NOB suppression that could result in long-term process stability and stable effluent quality for the subsequent downstream anammox treatment at mainstream municipal wastewater. The design strategy is herein demonstrated at the laboratory scale with synthetic wastewater; hence, the elevated TAN SALR strategy requires further investigation at the pilot scale using real wastewater with the potential to guide future implementation of a high-rate, compact, and low-operational intensive partial nitritation system at WRRFs.

In addition, regarding the future of the elevated loaded partial nitritation system following an anammox system as a two-stage configuration for effective nitrogen removal at mainstream municipal wastewater. Successful anammox operation as a two-stage PN/A system is achievable if operated with stable and steady partial nitritation effluent as well as an appropriate NO_2_^−^-N:NH_4_^+^-N metabolic ratio. According to the results from this study, the elevated loaded partial nitritation MBBR system is robust, effective, stable, steady and can maintain appropriate effluent ratios. As such, anammox enrichment and operation under mainstream conditions is feasible with the possibility of achieving high nitrogen removal rates and long-term process stability. Also, designing the partial nitritation MBBR system at an elevated TAN loading rate allows for high rate partial nitritation and, consequently, a small land footprint. Therefore, the introduction of the combined two-stage PN/A system can be achieved where insufficient land availability exists. On this note, further studies are proposed exploring the elevated loaded partial nitritation MBBR system as a two-stage PN/A system for enhanced nitrogen removal from mainstream municipal wastewaters.

## 4. Conclusion

The two-reactor in series elevated loaded mainstream partial nitritation MBBR system achieves stable and steady partial nitritation with an effluent NO_2_^−^-N:NH_4_^+^-N concentration corresponding to a molar stoichiometric ratio of 1.15:1. Hence, demonstrating possible suitable nitrogen speciation for the subsequent downstream anammox treatment. The biofilm thickness and morphology in the reactors show thick biofilms with the embedded biomass showing no viability constraint, as the embedded cells remained viable, supporting the observed stable and steady partial nitritation performance. The thick biofilm morphology likely reduced the diffusive transport of DO into the biofilm, that limits the DO uptake by NOB population. The AOB and NOB gene copy numbers of the MBBR biofilm show that rather than NOB population suppression, NOB activity suppression is the likely mechanism responsible for the stable and steady partial nitritation performance in the mainstream elevated loaded partial nitritation MBBR system.

## Data availability statement

The original contributions presented in the study are included in the article/supplementary material, further inquiries can be directed to the corresponding author.

## Author contributions

JI contributed to the conception and design of the study, performed the research, analyzed the data, and wrote the manuscript. HC contributed to data acquisition and analysis. RD developed the research questions and experimental design, supervised, and coordinated the research, reviewed the manuscript, and acquired funding for the research. All authors contributed to the article and approved the submitted version.

## Conflict of interest

The authors declare that the research was conducted in the absence of any commercial or financial relationships that could be construed as a potential conflict of interest.

## Publisher’s note

All claims expressed in this article are solely those of the authors and do not necessarily represent those of their affiliated organizations, or those of the publisher, the editors and the reviewers. Any product that may be evaluated in this article, or claim that may be made by its manufacturer, is not guaranteed or endorsed by the publisher.
